# Complexity Analysis of a Versatile Video Coding Decoder over Embedded Systems and General Purpose Processors

**DOI:** 10.3390/s21103320

**Published:** 2021-05-11

**Authors:** Anup Saha, Miguel Chavarrías, Fernando Pescador, Ángel M. Groba, Kheyter Chassaigne, Pedro L. Cebrián

**Affiliations:** Software Technologies and Multimedia Systems for Sustainability (CITSEM) Research Center, Universidad Politécnica de Madrid (UPM), 28040 Madrid, Spain; miguel.chavarrias@upm.es (M.C.); fernando.pescador@upm.es (F.P.); angelmanuel.groba@upm.es (Á.M.G.); ka.chassaigne@alumnos.upm.es (K.C.); pl.cebrian@alumnos.upm.es (P.L.C.)

**Keywords:** versatile video coding, complexity analysis, H.266, codec, multicore, heterogeneous, GPU, inter prediction, deblocking filter, adaptive loop filter

## Abstract

The increase in high-quality video consumption requires increasingly efficient video coding algorithms. Versatile video coding (VVC) is the current state-of-the-art video coding standard. Compared to the previous video standard, high efficiency video coding (HEVC), VVC demands approximately 50% higher video compression while maintaining the same quality and significantly increasing the computational complexity. In this study, coarse-grain profiling of a VVC decoder over two different platforms was performed: One platform was based on a high-performance general purpose processor (HGPP), and the other platform was based on an embedded general purpose processor (EGPP). For the most intensive computational modules, fine-grain profiling was also performed. The results allowed the identification of the most intensive computational modules necessary to carry out subsequent acceleration processes. Additionally, the correlation between the performance of each module on both platforms was determined to identify the influence of the hardware architecture.

## 1. Introduction

The increasing global availability of smart consumer electronic devices is causing a growing demand for full HD/ultra-HD videos, which is predicted to account for more than 80% [1] of internet traffic by 2022. However, the capacity of storage devices and communication channels is limited. As a result, it has become necessary to obtain better video compression techniques that can achieve bitrate improvements over high efficiency video coding (HEVC) [2] while maintaining quality.

Considering this situation, the new versatile video coding (VVC) standard was officially released in July 2020 [3] by the Joint Video Experts Team (JVET) of the ITU-T and JCT groups within ASO/IEC. Compared to its predecessor, the new standard has achieved a bitrate improvement of 50% [4] while maintaining quality.

Various new features have been adapted to VVC to achieve better coding efficiency [5]. For example, the largest size of coding tree units (CTUs) is 128 × 128 pixels, quadtree with nested multi-type tree (QTMT) for coding unit (CU) partition, multiple transform selection (MTS) support up to 87 intra coding modes [6], cross-component linear model, affine motion compensation [7] and adaptive loop filter (ALF). However, these features come with an increment in computational complexity that has been roughly evaluated as ×10 in the encoder and ×2 in the decoder [8]. Therefore, it has become necessary to analyse the complexity details of the VVC decoder to achieve real-time performance in embedded devices with limited resources. Several studies have presented complexity analyses of previous standards in [9,10,11,12,13,14]. The main target of a complexity analysis is to guide upcoming research towards acceleration of the reference software as it is focused on accuracy, completeness and dependability, but not in real-time performance of consumer electronics systems.

To date, a few VVC implementations have attempted to accelerate the reference software for real-time performance. Fan et al. proposed a pipelined 2D transform architecture for VVC discrete sine transform VII and discrete cosine transform VIII [15]. In [16], a general purpose processor (GPP)- and graphical processing unit (GPU)-based implementation of a VVC decoder was performed to accelerate the decoder by optimizing the motion compensation (MC). The proposed implementation led to a new partition of the coding unit, and organization of suitable thread schemes of the MC were implemented over the GPU. Moreover, the authors of the article [17] presented a parallelized multi-CPU core-based VVC decoder, for which a redesign of the decoding task had been proposed using load balanced task parallelization and data parallelization at the level of the CTU. In addition, various other studies have presented methods to accelerate partial functionalities of a VVC decoder over a GPU using OpenHEVC [18]. In [19], new adaptive multiple transforms of VVC were ported to an embedded GPU. Thus, a 2D transform was performed over a GPU, whereas the remaining decoding blocks were processed in a GPP.

This article begins with the premise that the increased complexity of new standard software solutions will require extensive optimisation processes. To ensure the success of these processes, it is important to characterise the computational load of the algorithms in detail. To this end, the authors of the paper decided to implement and characterise, on two different platforms based on general-purpose processors, the reference software VVC Test Model (VTM) in its version 8.0 [20]. The profiling process was divided into two stages: in the first one, the coarse-grained analysis was carried out. Here the main blocks of the decoding algorithm were featured. Then, the more computationally heavy blocks were analysed at a fine-grained level of detail. The test bench consists of six high-resolution sequences, using both 8 and 10 bit-depths, for two different configurations and four quantization parameter (QP) values. This work is detailed in this paper, introducing the following contributions:

1. An in-depth analysis of the new VVC standard to assist in the development of further and necessary optimisations. The more detailed analysis is addressed to inter prediction (IP), deblocking filter (DBF), and ALF blocks.
2. Two platforms, whose architectures are state of the art, were used:
  (a) An AMD-based high performance processor [21].
  (b) A heterogeneous NVIDIA Jetson AGX Xavier System on Chip (NXSoC) [22].
3. Possible correlations between the computational load of the algorithm blocks on each of the architectures were obtained and compared.
4. A comparison between the obtained results with different VVC implementations available in the scientific literature was performed.

The remainder of this paper is structured as follows: Section 2 summarizes the main functional blocks of the VVC codec. In Section 3, the working environment is described. In Section 4, the results of the performance analysis of the VTM decoder, inter prediction, DBF, and ALF over the platforms are shown. In Section 5, a comparative study of the complexity analysis of various VVC decoder implementations is presented in the discussion. Finally, Section 6 concludes the paper.

## 2. Versatile Video Coding (VVC)

**Figure 1:**
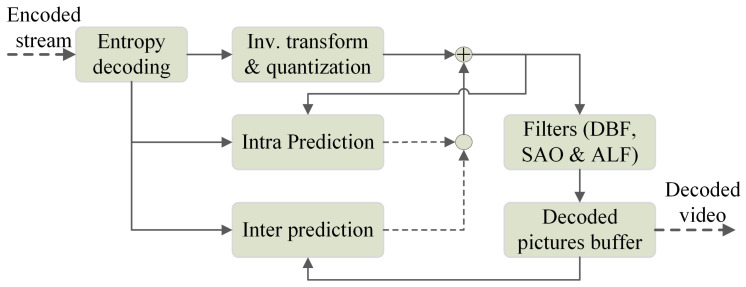
General block diagram of a VVC decoder.

The VVC codec is based on the same hybrid coding scheme used in previous standards, such as HEVC [2] or AVC [23]. The VVC decoding process scheme is shown in Figure 1 and can be summarized as follows: bitstream is the input, and video is the output of the system. First, the decoding process starts from entropy decoding of the bitstream. Here, context adaptive binary arithmetic coding (CABAC) [24] gives partitioning information of blocks named coding tree units using inter-picture prediction, intra-picture prediction, and coded residual data [4]. Therefore, to reconstruct the coded residual data, inverse quantization is applied followed by inverse transformations (primary and secondary). Finally, in-loop filters are used to process accumulative reconstructed residual and predicted data. The in-loop filter contains the DBF, the sample adaptive offset (SAO), and ALF filters. Next, the main modules integrating the decoding process are presented and detailed.

### 2.1. Picture Reconstruction

**Figure 2:**
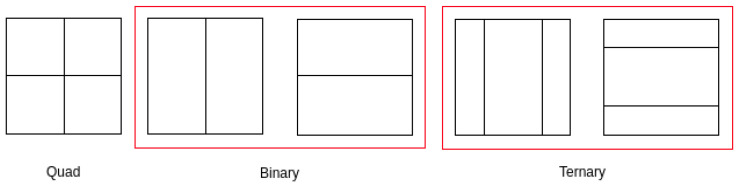
VVC supported partitioning schemes: quad, binary and ternary splitting structures.

For VVC, a similar but extended CTU concept was inherited from its predecessor, HEVC. In the new standard, the quadtree scheme had been replaced earlier by a quadtree plus binary tree [25]. Therefore, a vertical or horizontal directional partition of a binary tree was added to extend the quadtree plus binary tree. Finally, QTMT partitioning was adapted to the VVC standard. In the QTMT scheme, the blocks were divided into smaller square sub-blocks for the QT structure and into smaller rectangular binary and ternary sub-blocks for the multi type tree structure. Moreover, a block can be divided into the horizontal or vertical direction using binary and ternary splitting. Further, in the ternary split, a block was divided into three sub-blocks with a central sub-block 2 times larger than the outer blocks. The VVC supported partition schemes are displayed in Figure 2. In addition, VVC allowed a maximum CTU size of 128x128 pixels [26]. These features helped VVC achieve a more flexible and efficient partitioning structure compared to the HEVC standard.

### 2.2. Entropy Decoder

The entropy decoding process used in VVC is similar to that used in HEVC. However, the VVC CABAC engine is more advanced than its predecessor. Here, the index of the current state was used to linearly express the probability. Therefore, the pre-computed look-up table of HEVC was removed, and the multi-hypothesis probability update model was applied to improve the accuracy. In addition, the VVC CABAC introduced a QP dependent initialization model where total initialization values have 6-bit precision. In addition, VVC was dependent on the transform block size for the selection of coefficient group sizes. It allowed coefficient groups of 1 × 16, 2 × 8, 8 × 2, 2 × 4, 4 × 2 and 16 × 1.

### 2.3. Inverse Quantization (IQ) and Transform (IT)

In VVC, both the residual intra- and inter-CBs are encoded with an MTS scheme with three transform types: DCT–II, DCT–VIII and DST–VII. Rectangular H×W blocks are allowed with H≤64,W≤64 for DCT-II and H≤32,W≤32 for DCT–VIII and DST–VII (e.g., 4×64 or 32×16). To reduce the computational complexity of large transforms, the high frequency coefficients of H×64 and 64×W DCT-II transforms are *zeroed*; the same happens with the high frequency coefficients of the H×32 and 32×W DCT-VIII and DST-VII transforms. After this primary transform, a LFNST was applied before quantization. LFNST was added to VVC for performing further transform in low-frequency primary transform coefficients [27]. These coefficients are transform coefficients that come from directional intra prediction [28].

### 2.4. Intra Prediction

VVC introduced 65 directional intra prediction modes along with the planar and DC modes, whereas HEVC supported 33 directional intra prediction modes. In addition, a wide-angle intra prediction mode was adopted for non-square blocks by substituting some traditional angular modes, and the candidate with the most probable modes list was increased to 6. Other intra prediction coding tools were included in VVC: (1) The multiple reference line tool was used for angular prediction by referencing neighbouring lines 1 and 3 of the prediction block [29]. (2) The mode-dependent intra-smoothing tool was used to enhance the prediction efficiency using a four-tap intra filter. (3) A cross-component linear model was used in chroma samples for prediction to build reconstructed luma samples in the same CU.

### 2.5. Inter Prediction

The main contribution regarding inter picture prediction in VVC is the introduction of the merge mode along motion vector differences (MVD) that is used for finer representation of motion parameters and the adaptive motion vector resolution [8]. Similar to HEVC, the merge mode adopts candidates from spatial, temporal, and zero motion vectors. Further, it adopts a pairwise average vector, and a history-based vector [5]. VVC has achieved better precision accuracy than its predecessors by performing motion prediction at the sub-coding unit level. Moreover, affine motion compensated predictions [30] have been previously performed for handling zooming operations, rotation, and transformation motions. Here, two or three motion vectors were used to describe a sub-prediction unit. Therefore, triangle partitioning was applied at the CU level, where CU was divided into two triangles. This approach allowed more precise predictions within a CU.

### 2.6. In-Loop Filtering

The adaptive loop filter (ALF) is a new feature for VVC; the other filters, DBF [31] and SAO [32], show a high degree of similarity to those of HEVC. VVC has adopts DBF, SAO and ALF [33] to handle visible artefacts such as flickering, colour shift, blocking, blurring, and ringing in the reconstructed samples. In VVC, ALF two diamond-shaped filters are applied: one of 7 × 7 diamond shapes with thirteen different coefficients to luma blocks and the other of 5 × 5 diamond shapes with seven different coefficients to chroma blocks. Based on the vertical, horizontal, and 2 diagonal gradients, one of the twenty-five classes was chosen for each 4 × 4 block. Therefore, Wiener filters were computed for each class. However, before in-loop filtering was initiated, luma mapping with chroma scaling was applied to adjust the input luma signal.

## 3. The Working Environment

**Figure 3:**
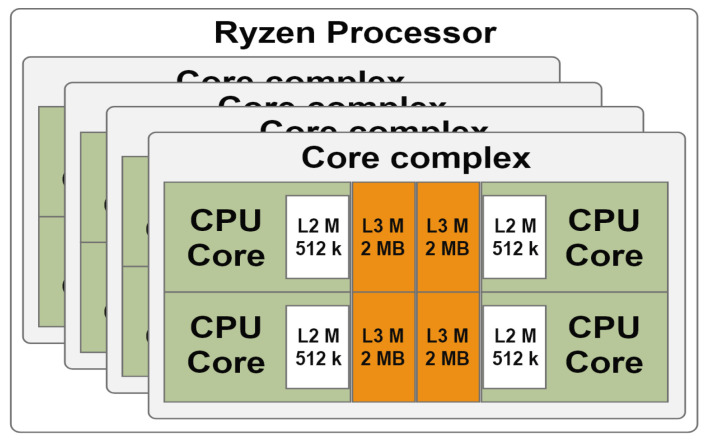
The architecture of a Ryzen processor.

In this study, two platforms were used: an AMD Ryzen Threadripper high performance processor [34] and an embedded NXSoC [22]. The architecture of the Ryzen processor is shown in Figure 3. This processor is based on Zen microarchitecture [35], which has a primary building block a core complex (CCX). Ryzen has 4 processing complexes, and each CCX includes 4 cores. Each core provides 2 threads with a base clock speed of 3.4 GHz, and all 32 threads have simultaneous multi-processing power. Each core is called a HGPP in this paper. In addition, all 4 cores share 8 MB of L3 cache memory, and each has its own 512 KB of L2 cache memory. All the L3 cache memories of different CCXs are allowed to be addressed and accessed by all CCX modules [36]. The maximum memory speed was 3.67 GHz.

**Figure 4:**
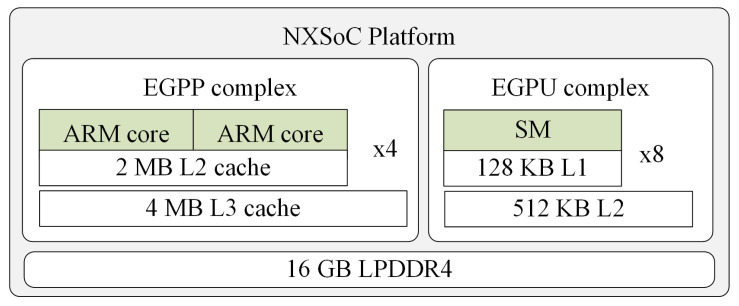
Representation of the architecture of the EGPP+EGPU platform, integrated by 8 EGPP cores, a GPU core with 8 SMs and shared DRAM.

The architecture of NXSoC is presented in Figure 4. In summary, it is composed of an EGPP complex and an Embedded GPU (EGPU) [37] complex, where the EGPP complex has 4 clusters and each cluster has 2 ARM cores with a maximum clock frequency of 2.26 GHz. Each of these cores is called an EGPP in this study. In a cluster, 2 EGPP cores share 2 MB L2 cache memory. Furthermore, all the clusters have access to 4 MB L3 cache memory.

The VTM8.0 reference software was ported over a HGPP and an ARM-based EGPP. Here, the CMake [38] toolchain was adapted for the EGPP. However, no modification was required for the HGPP. Then, “-O3” optimization of the GCC7.5 compiler was activated for both platforms. Therefore, profiling was obtained by executing the decoder using only one core of both the HGPP running at 3.40 GHz and EGPP running at 2.26 GHz. Here, only one core was used to fairly compare complexity analysis results obtained for both platforms.

## 4. Complexity Analysis of the VVC Decoder

In this section, the test bench and the collection of test sequences used are presented. A coarse grain profile of the VTM8.0 decoder is presented over the HGPP and EGPP. Four QP values were chosen for the test bench: 22, 27, 32 and 37. Here, the computational load of the main modules of the VTM8.0 decoder is outlined. Further, fine-grain profile of the most consuming modules, inter prediction, deblocking filter and adaptive loop filter modules over the HGPP and EGPP is shown.

### 4.1. Test Bench Description

The profiling was carried out by decoding two A1-Class video sequences, Camfire (CF) and FoodMarket4 (FM4); two A2-class sequences, DaylightRoad2 (DL2) and ParkRunning3 (PR3); and two B-Class sequences, BasketballDrive (BBD) and BQTerrace (BQT). These sequences were obtained from the test sequence set of common test conditions [39]. All sequences were encoded using the same VTM software version [20]. The features of the sequences are presented in Table 1. For each sequence the four QPs, two configurations, all intra (AI) and random access (RA), were used.

The VTM8.0 decoder was profiled to identify the most time-consuming functional blocks of the decoder and instrumented to include timestamps before and after each call to the following functional blocks: entropy decoding (ED), inverse transforms (TX), inter prediction (EP), intra prediction (IP), deblocking filter (DBF), and adaptive loop filter (ALF). The difference among the total times to decode the entire sequence and the sums of the times measured in all functional blocks were assigned to other (OT). Notably, SAO filtering had a reduced impact on the total load (below 3% in all cases). For simplicity, in some tables, SAO impact is included in the OT part. In these cases the SAO is not independently shown.

**Figure 5:**
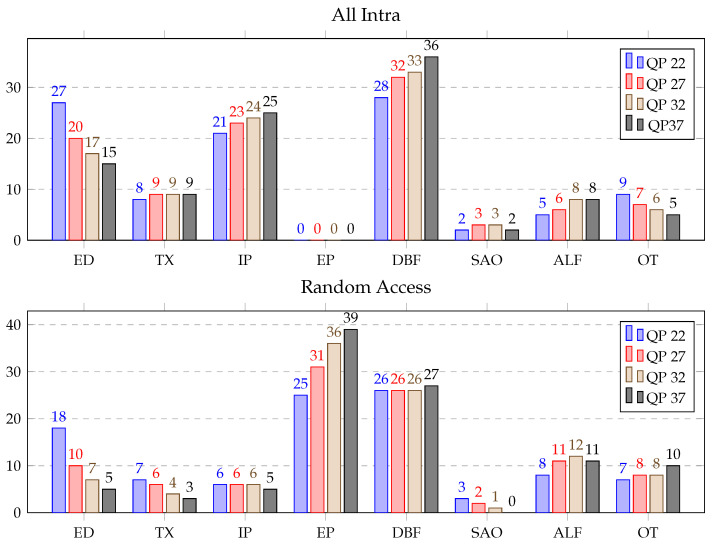
Average time distribution for different blocks of the VVC decoder (in %) over the HGPP.

Table 2 illustrates the results obtained over the HGPP with details of consumed time (in secs). The average computational load (in %) consumed by the different blocks of the VVC decoder over the HGPP is displayed in Figure 5. As shown in this figure, in-loop filers were the most demanding blocks for AI configurations, which represented approximately average 40% of the decoding time for DBF, SAO and ALF. Entropy decoding and intra prediction accounted for approximately average 20% and 23%, respectively. Furthermore, the time consumed by IP, DBF, and ALF were increased by 1–4% with the increment of the four QP values from 22 to 37. However, the scenario was opposite for ED and OT which decreased by 1–7%. TX and SAO consumed similar time for different QPs. Therefore, for the RA configurations, the most demanding blocks were inter prediction and the in-loop filters, which consumed approximately average 70% of the decoding time. Entropy decoding and inverse transforms accounted for approximately average 9% and 5% of the decoding time, respectively. Further, IP and DBF consumed average 6% and 26% of the decoding time for all the QPs. Moreover, the computational cost of the ED, TX, and SAO were decreased, and the computational cost of the EP, ALF, and OT were increased with the changes of the four QPs from 22 to 37. In addition, 0.6 to 17 frames per second (fps) ratios were achieved for different configurations and QPs over the HGPP.

The VVC decoder profile over the EGPP is shown in Table 3. Here, the average decoding times (in secs) consumed by each functional block are presented for each configuration and QP over the EGPP-based architecture. These results indicate that the system was able to decode between 0.3 and 5 fps, depending on the sequence, QP and configuration. Therefore, to accomplish real-time performance, an increase in fps of roughly ×100 would be needed. With our experience working on the optimization of previous video decoders with HEVC-based implementations, a speedup of up to ×10 could be obtained by both optimizing the code and executing it over several cores (e.g., the 8 cores in the NXSoC) [40]. Hence, an additional minimum speedup of ×10 should be obtained by GPU-based accelerators to achieve real-time performance.

**Figure 6:**
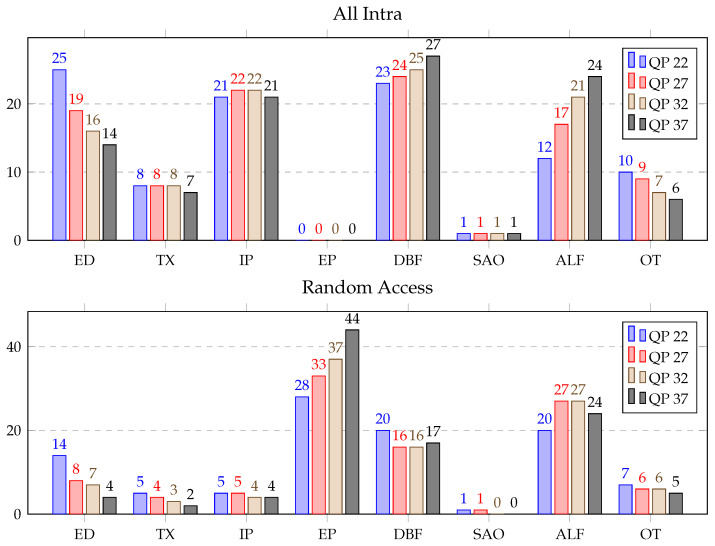
Average time distribution for different blocks of the VVC decoder (in %) over the EGPP.

In addition, Figure 6 shows the computational cost (in %) for each functional block, QP and configuration, averaged for all the test sequences. The most time-consuming modules for AI configurations were the in-loop filters, the intra prediction and the entropy decoding blocks, which accounted for approximately average 44%, 22%, and 19% of the decoding time, respectively. Further, TX, IP, and SAO consumed similar time for different QPs. In addition, the computation load of ED and OT decreased and the computation load of DBF and ALF increased for the increment of the four QPs from 22 to 37. On the other hand, inter prediction and in-loop filtering blocks were the most time-consuming blocks for the RA configuration, and they accounted for approximately 78% of the decoding time. Moreover, the computational cost of all the decoder blocks were decreased for the changes of four QPs from 22 to 37 except the computational cost of EP and ALF.

The implementation of the VVC 8.0 decoder over the HGPP and EGPP showed that the ALF consumed more computational time than the DBF in the EGPP. However, the scenario was opposite for the HGPP. The other modules had similar shares of decoding time for the HGPP and EGPP. This analysis points out where optimization efforts can be overtaken. In the following Section 4.3, Section 4.4 and Section 4.5, we delve deeper in the details by presenting a detailed profiling of the IP, DBF, and ALF blocks, respectively.

### 4.2. Correlation between the HGPP and EGPP

In this subsection, the correlation between the HGPP and EGPP is presented. This analysis allowed us to identify the effect of the hardware resources on the performance of different decoding blocks. Table 4 presents the average processing times (in secs) of each decoding block over the HGPP and EGPP for the AI and RA configurations. The processing times of ED, TX, IP, DBF, and OT were roughly ×2 higher than the processing time of the EGPP with respect to the HGPP for both the AI and RA configurations. Moreover, the processing times of the ALF were ×7.1 and ×7.4 higher than those of the EGPP for the AI and RA configurations, respectively. Additionally, for the RA configurations, the EGPP required ×3.5 more time to process the EP. However, the fps values obtained over the HGPP for the AI and RA configurations were ×2.5 and ×3.3, respectively. These results show that the influence of the architecture impacts mainly the EP block for the RA configurations and the ALF block for the AI and RA configurations. Therefore, the acceleration process is addressed in the EP and ALF over the EGPP.

### 4.3. Complexity Analysis of the Inter Prediction

In this section, the detailed profiling of inter prediction is presented using all the sequences with the RA sequences over the HGPP and EGPP. In the decoder profiling, timestamps were placed before and after the following inter prediction modules: inter texture (ITEX), sub-prediction unit MC (SPUM), sub-prediction unit bio (SPUB), uni-directional prediction (UDP), decoder side motion vector refinement (DMVR), weighted prediction (WP). In turn, other (OT) part was calculated as the difference between the global EP measurement and the sum obtained from previous modules.

Table 5 represents the average consumed time distribution (in %) for different parts of the inter prediction over the HGPP and EGPP. The DMVR and UDP were the most computationally heavy modules in the inter prediction for both GPPs. Both DMVR and UDP together accounted for more than average 58% of the total time required for the inter prediction. The other modules consumed amounts individually ranging from 1% to 28% of the total time. The total average time distribution presents a broader view of the inter prediction profiles.

**Figure 7:**
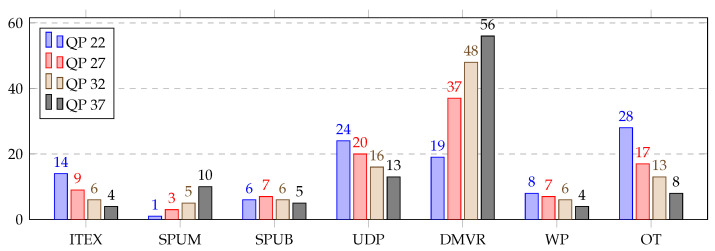
Average time distribution for different parts of the inter prediction (in %) over the HGPP.

A detailed view of the inter prediction over the HGPP is shown in Figure 7, which shows the average time distribution for different parts of the inter prediction (in %) over the HGPP with QP 22, 27, 32 and 37. It was found that with the change in QP from 22 to 37, the average computational load of the DMVR increased from 19% to 56%, and the average computational load of the UDP decreased from 24% to 13%. The average computational load for the other inter prediction parts varied by 1% to 11% for different QP values.

**Figure 8:**
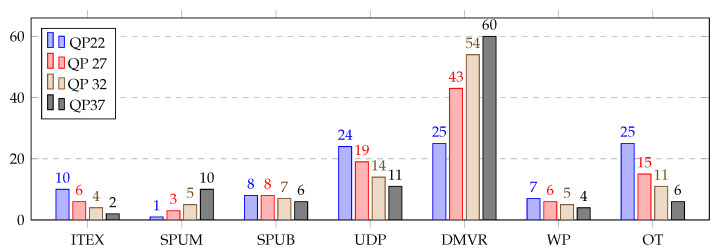
Average time distribution for different parts of the inter prediction (in %) over the EGPP.

The inter prediction profiling results over the EGPP are shown in Figure 8, where the average time distribution for the different parts of the inter prediction (in %) with QP 22 to 37 is presented. Here, the DMVR and UDP functions followed the same pattern as the profiling of the inter prediction over the HGPP. The average computational load of the DMVR increased by 35%, and the average computational load of the UDP decreased by 13% with the change in QP from 22 to 37. However, the DMVR represents approximately 5% more average computational load over the EGPP than over the HGPP, while the average computational loads for the other functions were slightly lower, except for the SPUM function.. Finally, considering this fine-grained analysis, the EP profiling results show that the DMVR and UDP are the most interesting functions to consider for acceleration, regardless of which platform is targeted.

### 4.4. Complexity Analysis of the Deblocking Filter

**Figure 9:**
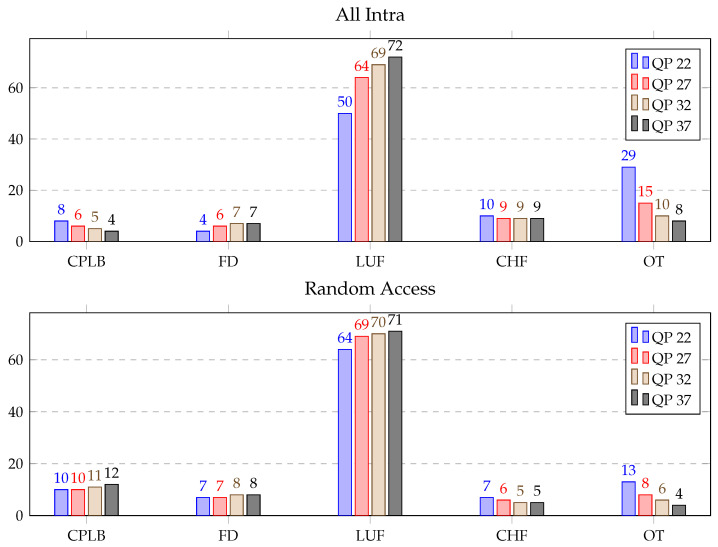
Average time distribution for different parts of the deblocking filter (in %) over the HGPP.

**Figure 10:**
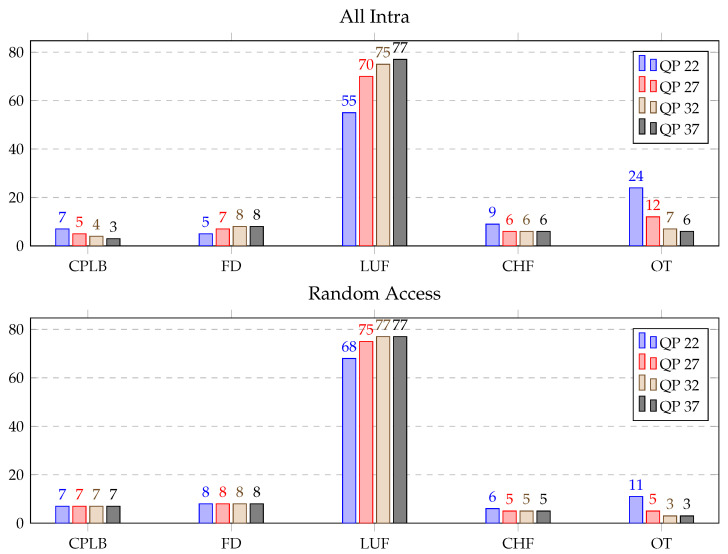
Average time distribution for different parts of the deblocking filter (in %) over the EGPP.

DBF consists of calculate position and length of the boundaries (CPLB), filtering decision (FD), luma filter (LUF), chroma filter (CHF) and other (OT) functions. Timestamps were set before and after these functions for detailed profiling of the DBF using the six sequences mentioned with respect to the test bench (Section 3B). In Table 6, the average time distribution (in %) for different parts of the DBF over the HGPP and EGPP is illustrated. It can be seen that the LUF function alone consumed from approximately 64% to 74% of the DBF time for the AI and RA configurations over the HGPP and EGPP. The other functions individually consumed from roughly 5% to 16% of the total DBF time. The details of the profiling of the DBF over the HGPP and EGPP are displayed in Figure 9 and Figure 10, respectively. Here, the average time distribution for different parts of the deblocking filter (in %) with QP 22, QP 27, QP 32 and QP 37 is presented.

Some general conclusions about DBF profiling can be outlined by comparing all the configurations of the sequences. The time spent by the LUF function for the AI configuration increased by 22% when the QP changed from 22 to 37 over both the platforms. For the RA configuration, the increment of the spent time was 7% and 9% over that of the HGPP and EGPP, respectively. Furthermore, the average computational load of the CPLB, FD and CHF functions remained similar for RA configurations over the EGPP. However, the average time consumed by OT decreased approximately 20% and 8% for the AI and RA configurations with the change in QP from 22 to 37 over the HGPP and EGPP.

The comparison study of DBF profiling over GPPs shows that the computational importance of LUF increased by approximately average 6% and that the computational impact of the CHF, FD, CPLB and OT varied by approximately 1% to 5% over the EGPP, than compared to the HGPP. In conclusion, and considering the detailed results presented in Figure 9 and Figure 10, LUF can be considered a potential candidate for optimization, as it is also the most time-consuming function of the DBF. Other potential candidates are CHF and CPLB.

### 4.5. Complexity Analysis of the Adaptive Loop Filter

The ALF is one of the most intensive computational modules of the decoder, and it consumed, on average, 16% of the total decoding time and 37% of the in-loop filtering time. It was composed of the following most relevant sub-modules: copy reconstructed YUV (CRY), virtual boundaries check (VBC), derivative classification (DeC), luma component filtering (LCF), chroma component filtering (CHCF), cross-component filtering (CRCF) and others (OT). ALF profiling was performed in the same fashion as for the DBF.

The ALF profiling results are shown in Table 7, where average processing times (in %) for ALF block profiling over the HGPP and EGPP are presented. The most time-consuming functions of the ALF over the HGPP were for LCF, with 37.7% of the total time consumed for the AI sequences and 43.2% for the RA sequences, and CRCF, with 22.9% of the total time consumed for the AI sequences and 11.6% for the RA sequences. The percentage of computational load of the DeC was 17.5% for the AI sequences and 21.6% for the RA sequences, and the computational load of the CHCF was 10.7% for the AI sequences and 8.0% for the RA sequences. However, ALF profiling over the EGPP revealed that the LCF, CHCF and DeC functions consumed approximately 90% of the total time, where LCF alone consumed approximately 70% of the time used for the ALF process.

**Figure 11:**
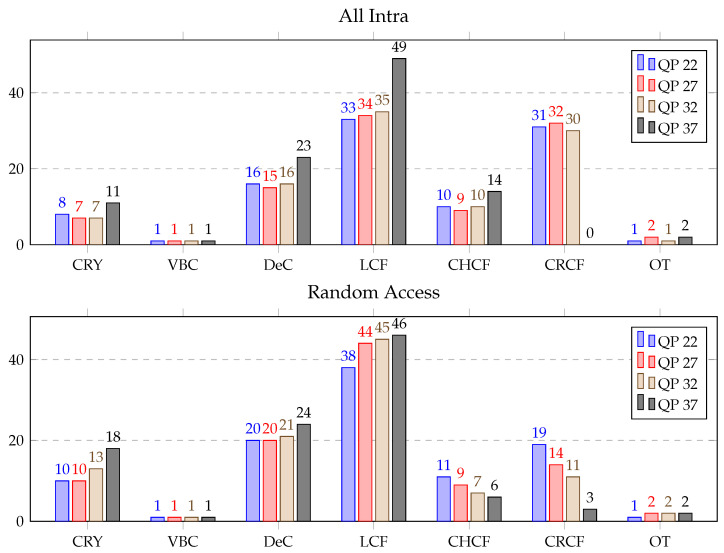
Average time distribution for different parts of the adaptive loop filter (in %) over the HGPP.

Detailed ALF profiling for QPs 22 to 37 with all the configurations over HGPP is displayed in Figure 11. The average computational load of the CRY, DeC, LCF, CHCF, and CRCF varied maximum 2% for the AI sequences with QPs 22, 27 and 32. But for QP 37, CRY, DeC, LCF, and CHCF increased 3% to 14% with respect to other QPs and CRCF decreased to 0%. Moreover, all the ALF functions for the RA sequences showed a pattern similar to that of the ALF function for the AI sequences. However, the time consumed by the CHCF and CRCF decreased by 5% and 16%, respectively with QP 37 compared to QP 22.

**Figure 12:**
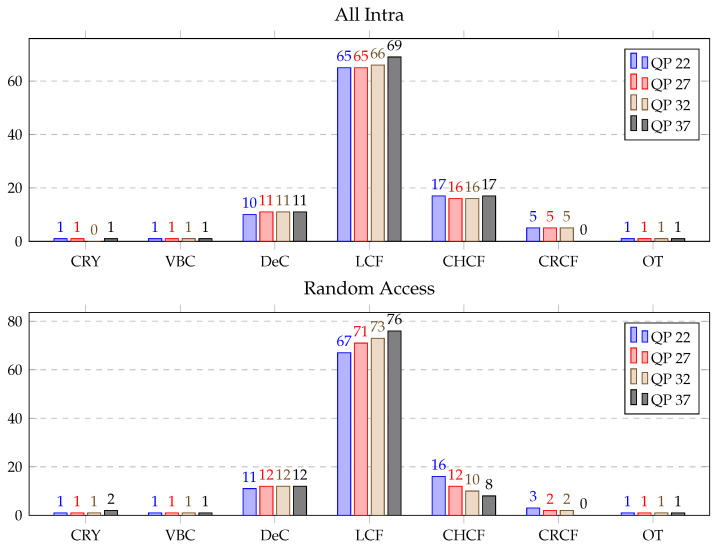
Average time distribution for different parts of the adaptive loop filter (in %) over the EGPP.

Figure 12 presents the average time distribution for different parts of the ALF (in %) over the EGPP. The time consumed by all the functions remained the same or varied slightly, less than 4% for all cases, with the change in QP. Therefore, it can be summarized that the existence of a large difference in the average processing time distribution of different submodules of ALF is observed between HGPP and EGPP. It can be stated that different strategies can be used to accelerate EGPP, mainly focusing on the acceleration in LCF, DeC, and CHCF functions.

## 5. Discussion

The new video coding standard VVC introduces numerous advantages in video processing technology. However, it also brings increased computational complexity compared to its predecessor. In this section, a comparative study of the complexity analysis of our HGPP- and EGPP-based implementations of VTM8.0 with other VVC decoder implementations is presented.

The maximum and minimum numbers of fps are compared for different implementations of the VVC decoder in Table 8. To fairly compare, only solutions from the reference coding software for A-class resolution sequences with the QP 22, 27, 32 and 37 were included in this section. To the best of our knowledge, no other VVC implementations based on a source code different from VTM8.0 have been reported in the scientific literature.

In [17], the complexity analysis of the VTM5.0 decoder was illustrated using a core i9-9900X HGPP with a clock speed of 3.5 GHz. This implementation decoded between 1.3 and 4.2 fps for RA sequences of A-Class resolution with four QP values 22 to 37.

A HGPP core of an i7-8700K 4.7 GHz processor was used in [16] to analyse the complexity of a decoder based on VTM6.1. In this previous study, A-class sequences of RA configurations with QPs: 22, 27, 32, 37 were used as test benches. Additionally, an optimized complexity analysis was reported using RTX 2080Ti 1.6 GHz GPU. Decoding ratios of 1.7 to 4.7 fps and of 2.1 to 8.4 fps were obtained for unoptimized and optimized decoder versions, respectively. In [5], a core i7-4790K 4.0 GHz HGPP was used to profile an implementation of a VTM6.0 decoder, but the global fps or processing times were not reported in this paper. As a result, it would not have been possible to make a fair comparison in terms of fps, but later in this section, it is used to compare the computational load among functional blocks.

In our study, HGPP- and EGPP-based VTM8.0 complexity analyses were carried out. A-class sequences with four QP values 22, 27, 32, 37 were used, obtaining ratios between 0.6 and 2.3 fps, and between 1.2 and 4.1 fps over the HGPP, respectively, for the AI and RA configurations. In addition, decoding ratios of 0.3 to 0.8 fps and from 0.4 to 1.2 fps were obtained over the EGPP for the AI and RA configurations, respectively. The sequence with minimum-maximum (min-max) fps over the HGPP was similar to that of [17] for RA configurations, as both implementations used an HGPP with similar clock speeds. In addition, the min-max fps of [16] for RA configurations was approximately ×1.2 than our HGPP implementation, as the clock speed of the HGPP used in the previous study implementation was ×1.4 than our HGPP. Moreover, the GPU-based optimized solution of the previous study obtained almost double the min-max fps obtained with our HGPP implementation.

In summary, the obtained fps for the RA configurations over all HGPP-based implementations varied slightly due to the different processor resources, which included clock speed or cache memory, among others. Additionally, an approximately double increase in speed was achieved using a GPU. However, for RA configurations, fps obtained over HGPP with respect to EGPP was roughly ×3. These results will guide future work towards real-time decoding over embedded platforms.

In Table 9, a comparison of the computational load distribution for different blocks of the decoder is shown. Here, the obtained results are compared with [5,16,17]. For both, RA and AI configurations, it is evident that our results for HGPP and EGPP were only significantly different with respect to the DBF and ALF blocks. In the HGPP, the DBF load was approximately 9% higher than that in the EGPP, while the ALF load showed the opposite trend and an average increase of 14% in the EGPP compared to the HGPP. This indicates that the architectural resources of the EGPP mainly affected the ALF block. Therefore, acceleration efforts should be carried out in the ALF for the EGPP implementation.

For the RA configuration, our HGPP results on the decoding process were similar to those reported in [5]. However, it is interesting that the DBF load was increased by approximately 10% in our HGPP analysis. Additionally, for the AI configuration, the computational load distribution of [5] was similar for the TX and SAO, roughly ×1.5 for the ALF, and approximately ×0.5 for the IP and DBF with respect to our HGPP implementation. Moreover, the results presented by [16] using VTM6.1 corresponded approximately to those obtained by our EGPP implementation, with the main difference again with the DBF, being 8%. Furthermore, the computational load of all the decoding blocks presented in [17] was approximately 6% on average, varying from our HGPP implementation.

These analyses imply that our HGPP architecture mainly affects DBF blocks with respect to the architecture used in [5,16]. Additionally, the architectural influence between our HGPP and the HGPP used in [17] varies. However, the architectural resources of our HGPP processor mainly affect DBF blocks with respect to the processor used in [17]. Moreover, our EGPP processor has a greater impact on DBF blocks than the processor used in [16].

### 5.1. Towards the Acceleration Process

In this section, an estimation analysis is performed on the results obtained in Section 4. This estimation was conducted as follows: first, recent works presenting contributions on software-level optimization-based approaches were considered. Second, hardware platform-based solutions were also analysed. Finally, a summary, collecting both approaches, is presented.

#### 5.1.1. Estimation Based on Software Level Optimization

The work presented by Gudumasu et al. [17] reported a software-based parallelization of the VVC decoder, which was analysed for software-level estimation analysis. To the best of our knowledge, this was the unique published work proposing software-based optimizations with the new VVC standard. Here, the VTM5.0 decoder was redesigned using load balancing task parallelization (LBTP) and CTU-based data parallelization (CDP). The LBTP was applied to the CABAC and slice decoding task, whereas CDP was applied to each sub-module of the slice decoding task. This implementation achieved an average processing time reduction of 70.2% for motion vector component derivation, 88.2% for inter-reconstruction, 68.9% for intra-CU reconstruction, 86.8% for the inverse re-shaper, 90.2% for the DBF, 72.3% for the SAO, and 88.2% for the ALF using 4k sequences of A-class resolution, similar to those stated in Section 4.1. The aforementioned ratio improvements of different VVC decoder blocks were used to calculate the performance of the corresponding decoder block over HGPP- and EGPP-based implementations.

#### 5.1.2. Estimation Based on Hardware Platforms

Han et al. [16] presented a GPU-based motion compensation optimization of a VVC decoder. This study was chosen for estimation analysis over heterogeneous platforms because it performed acceleration of a VTM6.1 decoder using an architecture similar to that stated in Section 3. Hither, all the decoder blocks, including entropy decoding, intra prediction, inverse quantization/transformation, and in-loop filter, were executed on a CPU, and the execution of the motion compensation module was migrated to a GPU. This implementation achieved a 16-fold times acceleration compared with the performance on a CPU using RA sequences of an A-Class resolution similar to those mentioned in Section 4.1. In addition, Vázquez et al. migrated the new VVC adaptive multiple transform (AMT) over an EGPU in [19]. In this implementation, the 2D transform was split into one 1D vertical transform and one 1D horizontal transform. On the other hand, the execution order was a vertical transform followed by a horizontal transform. This study achieved an 11-fold improvement in the TX module time. Therefore, the aforementioned improvements of 16% and 11% acceleration for the EP and TX, respectively, were applied to the results obtained in Section 5.1.1.

#### 5.1.3. Summary of Both Estimations

Finally, regarding both the detailed profile of the reference VVC software, and the optimizations applied over similar solutions, a rough estimation of the speed-up performance is presented. To complete this study, the following aspects were considered: the distribution of the measured computational load for each decoder block, the impact of the reviewed optimizations on each of these blocks, and the resulting speed-up factor, for each platform and configuration of the sequences, from an optimistic point of view and from a pessimistic perspective. Table 10 summarizes this study. Here, approximate minimum and maximum performances obtained with current solutions and with the presented test bench are shown. Two speed-up factors, one pessimistic and one optimistic, were calculated taking into account both, the profiling results presented in this work, and the acceleration techniques applied in these algorithms. The estimation of the performance that can be obtained in an optimized version of the decoder was then made taking into account the parameters of the study. As a preliminary conclusion, real-time decoding would not be obtained for almost all cases. In this situation, both, hardware platform-based optimizations of different parts of the algorithm, and better performance ratios with the initial solution need to be accomplished to achieve real-time performance.

## 6. Conclusions

The new VVC standard comes with various advanced features compared to existing video codecs. However, these features come with higher computational complexity. In this study, the complexity analysis of a VVC decoder was revealed. In this presentation, a VTM8.0 decoder was instrumented and implemented over two different architectures: a homogeneous multicore HGPP integrated in an AMD processor and a heterogeneous ARM+GPU embedded platform. The goal of this work was to guide future work focusing on acceleration processes over the brand new state-of-the-art codec, especially over embedded systems and platforms with limited computational resources. First, coarse-grain profiling was implemented. Here, the computational load of the main coding blocks, ED, TX, IP, EP, and filters, was outlined. The coding blocks consuming the most time, the EP, DBF, and ALF, were then fine-grained analysed. The computational load distributions outlined where optimization efforts should be carried out. Moreover, these results were compared with the available implementation of VVC over other architectures, which showed that the influence of processor hardware architecture greatly affected the ALF and DBF blocks. In this work, the correlation between HGPP and EGPP was also calculated, and it was shown that the processing time of all decoder blocks was roughly ×2 over the EGPP with respect to the HGPP except for the ALF block, which was approximately ×7 greater, and the EP block, which was ×3.5 greater. In addition, the performance results showed ×2.5 and ×3.3 fps ratios running the decoder over the HGPP compared to the EGPP for the AI and RA configurations, respectively. Finally, a rough estimation analysis was presented based on software-level optimization and hardware platforms. It can be foreseen that an average improvement of ×4.8 and ×5.2 in decoding time could be achieved using the estimation analysis over the HGPP and EGPP, respectively.

## Figures and Tables

**Table 1:** Features of the VVC Bitstreams used in the tests.

Class	Sequence	Resolution	Frames	Bit Depth
A1	Camfire	3840 × 2160	300	10
	FoodMarket4	3840 × 2160	300	10
A2	DaylightRoad2	3840 × 2160	300	10
	ParkRunning3	3840 × 2160	300	10
B	BasketballDrive	1920 × 1080	500	8
	BQTerrace	1920 × 1080	600	8

**Table 2:** Processing times (in s) for each decoding block and global fps achieved over the HGPP.

	Seq.	QP	ED	TX	IP	EP	DBF	ALF	OT	fps
All Intra	CF	22	105.1	24.8	77.4	0.0	111.6	14.6	49.5	0.8
		27	65.1	21.6	66.3	0.0	96.1	15.5	35.6	1.0
		32	31.7	16.1	49.3	0.0	75.0	16.8	18.2	1.5
		37	17.3	12.0	36.9	0.0	53.1	11.3	7.6	2.2
	FM4	22	41.0	28.1	61.5	0.0	80.1	16.1	11.8	1.3
		27	28.4	24.2	52.8	0.0	68.7	17.4	6.6	1.5
		32	19.4	19.6	43.2	0.0	56.6	16.3	4.0	1.9
		37	14.1	16.5	37.0	0.0	48.0	12.3	0.6	2.3
	DR2	22	155.9	36.7	93.5	0.0	151.8	19.2	58.1	0.6
		27	49.8	23.0	62.5	0.0	101.6	19.5	30.5	1.1
		32	31.6	16.9	47.0	0.0	80.1	18.9	20.8	1.4
		37	20.4	13.3	36.6	0.0	62.1	12.0	11.2	1.9
	PR3	22	107.8	38.1	88.1	0.0	100.8	19.2	29.8	0.8
		27	80.6	35.8	84.5	0.0	105.1	20.6	28.1	0.9
		32	55.9	29.7	72.4	0.0	95.8	20.3	22.9	1.0
		37	39.5	23.0	58.2	0.0	89.4	14.4	18.9	1.2
	BBD	22	52.2	15.0	40.0	0.0	51.5	8.9	25.7	2.6
		27	29.1	12.1	31.7	0.0	41.4	9.1	16.7	3.6
		32	18.3	9.3	24.5	0.0	31.2	8.4	10.2	4.9
		37	12.6	7.3	19.3	0.0	25.4	6.4	6.4	6.5
	BQT	22	99.3	20.0	56.9	0.0	65.1	9.6	44.3	2.0
		27	55.0	15.8	45.1	0.0	61.0	11.0	32.0	2.7
		32	35.4	11.5	34.4	0.0	48.0	10.4	23.6	3.7
		37	22.3	8.3	25.9	0.0	32.9	6.2	15.3	5.4
Random Access	CF	22	45.0	16.3	32.1	27.1	64.5	14.1	21.8	1.4
		27	17.0	10.3	20.2	22.0	43.4	13.5	13.0	2.2
		32	9.4	7.0	14.1	20.8	33.1	11.0	7.3	2.9
		37	6.5	4.6	10.0	20.0	30.2	11.0	8.4	3.3
	FM4	22	10.1	9.7	5.4	41.8	29.9	14.3	4.9	2.6
		27	6.3	7.1	4.2	42.8	25.8	13.0	4.6	2.9
		32	3.7	5.1	3.3	39.1	20.0	9.0	3.8	3.6
		37	2.6	3.4	2.6	36.4	18.1	5.9	5.0	4.1
	DR2	22	27.1	9.4	9.8	40.8	47.8	14.8	20.3	1.8
		27	8.2	4.1	4.9	39.9	29.6	12.6	11.4	2.7
		32	4.1	2.3	2.8	38.2	22.6	10.2	8.2	3.4
		37	3.1	1.4	2.0	37.1	21.6	8.0	9.4	3.6
	PR3	22	45.4	15.3	8.3	67.0	66.2	20.1	21.0	1.2
		27	23.2	9.3	6.9	58.6	49.9	19.6	17.2	1.6
		32	12.8	5.7	5.5	50.2	39.4	18.5	13.4	2.1
		37	6.5	3.3	4.0	42.7	29.2	13.0	9.8	2.8
	BBD	22	13.1	4.8	5.6	16.8	17.2	6.1	8.2	7.0
		27	5.8	2.9	3.7	15.2	12.2	5.4	5.0	10.0
		32	3.2	1.9	2.5	14.1	9.8	3.4	3.9	12.6
		37	2.1	1.2	1.7	13.5	8.9	3.9	3.8	14.2
	BQT	22	30.8	7.6	2.8	31.5	31.6	7.8	17.7	4.6
		27	6.4	1.9	2.0	16.2	11.4	6.2	7.8	11.6
		32	2.7	0.9	1.4	14.5	7.6	5.2	5.2	16.0
		37	1.8	0.5	1.0	14.3	7.3	4.3	5.3	17.4

**Table 3:** Processing times (in secs) for each decoding block and global fps achieved over the EGPP.

	Seq.	QP	ED	TX	IP	EP	DBF	ALF	OT	fps
All Intra	CF	22	240.3	58.4	188.7	0.0	233.3	116.7	126.3	0.3
		27	146.4	46.5	149.4	0.0	192.3	118.1	82.7	0.4
		32	69.7	34.3	109.2	0.0	133.1	117.9	37.7	0.6
		37	46.0	27.2	88.2	0.0	126.6	114.2	21.5	0.7
	FM4	22	99.3	57.9	139.4	0.0	174.5	120.2	35.3	0.5
		27	64.9	47.4	115.7	0.0	130.6	122.9	20.7	0.6
		32	46.5	39.1	98.2	0.0	111.5	121.6	12.0	0.7
		37	33.9	33.1	83.0	0.0	104.9	115.3	3.7	0.8
	DR2	22	328.6	82.5	224.0	0.0	302.0	118.1	141.4	0.3
		27	124.1	51.0	149.3	0.0	226.6	123.6	71.2	0.4
		32	81.6	37.3	110.9	0.0	150.5	122.6	45.0	0.6
		37	53.0	28.4	86.6	0.0	138.3	115.6	27.0	0.7
	PR3	22	228.3	86.9	209.2	0.0	220.6	123.4	78.0	0.3
		27	174.0	74.9	189.7	0.0	203.3	125.4	71.3	0.4
		32	129.8	62.8	165.8	0.0	205.5	125.4	58.8	0.4
		37	91.7	49.5	134.3	0.0	178.5	114.8	40.5	0.5
	BBD	22	117.7	35.4	99.3	0.0	85.6	50.6	59.0	1.1
		27	67.9	25.5	72.1	0.0	66.4	52.3	38.4	1.6
		32	44.8	19.5	55.4	0.0	53.2	51.5	22.6	2.0
		37	30.5	15.9	45.5	0.0	46.9	48.4	14.0	2.5
	BQT	22	224.1	50.0	146.2	0.0	128.6	58.3	115.7	0.8
		27	125.1	35.4	105.4	0.0	94.4	62.5	72.3	1.2
		32	84.6	26.5	81.9	0.0	78.4	62.4	52.3	1.6
		37	60.1	20.1	63.7	0.0	66.3	57.7	36.4	2.0
Random Access	CF	22	105.8	41.1	80.4	93.5	181.6	103.5	52.1	0.5
		27	41.5	23.2	49.4	64.0	79.5	95.8	23.3	0.8
		32	24.0	15.5	34.3	62.0	62.7	83.3	16.9	1.0
		37	14.2	10.3	24.5	63.3	58.8	78.4	15.4	1.1
	FM4	22	22.8	19.8	12.7	138.0	61.0	115.3	9.3	0.8
		27	9.5	10.8	9.7	138.5	45.2	99.7	16.2	0.9
		32	9.3	10.0	7.7	138.8	44.1	74.1	9.6	1.0
		37	6.4	6.8	6.0	135.2	39.6	46.8	10.9	1.2
	DR2	22	64.3	23.8	24.9	145.1	104.3	115.0	41.3	0.6
		27	21.8	9.5	12.0	138.8	55.6	104.6	22.6	0.8
		32	11.4	5.3	7.2	140.1	50.6	85.5	17.7	0.9
		37	6.9	3.2	4.9	139.3	46.2	54.6	16.5	1.1
	PR3	22	105.8	39.5	20.7	233.6	175.4	123.0	47.4	0.4
		27	61.8	24.1	17.1	200.3	106.0	122.3	40.9	0.5
		32	35.0	14.3	13.5	177.0	81.6	119.8	31.7	0.6
		37	17.7	8.1	10.0	163.9	72.8	110.6	23.1	0.7
	BBD	22	33.5	13.0	15.2	62.5	38.1	48.2	19.6	2.2
		27	15.9	7.2	9.4	54.0	24.3	43.2	12.3	3.0
		32	9.1	4.6	6.4	51.3	19.7	35.3	9.1	3.7
		37	5.3	3.0	4.5	53.5	18.4	28.7	7.6	4.1
	BQT	22	77.0	22.8	7.6	116.1	65.6	58.1	38.3	1.6
		27	16.5	4.8	5.4	57.0	22.7	51.7	16.9	3.4
		32	7.4	2.2	3.6	54.5	15.1	41.7	10.9	4.4
		37	4.4	1.3	2.7	58.8	14.2	28.4	9.4	5.0

**Table 4:** Average processing times (in secs) for each decoding block, average global fps achieved and ratio between HGPP and EGPP.

		ED	TX	IP	EP	DBF	ALF	OT	fps
AI	HGPP	49.5	19.9	51.9	0.0	72.2	13.9	22.0	2.2
	EGPP	113.0	43.6	121.3	0.0	143.8	98.3	53.6	0.9
	Ratio	2.3	2.2	2.3	n/a	2.0	7.1	2.4	2.5
RA	HGPP	12.4	5.7	6.5	31.7	28.2	10.5	9.7	5.6
	EGPP	30.3	13.5	16.2	111.6	61.8	77.8	21.6	1.7
	Ratio	2.5	2.4	2.5	3.5	2.2	7.4	2.2	3.3

**Table 5:** Average processing times (in %) for each inter prediction block profiled over the HGPP and EGPP.

	ITEX	SPUM	SPUB	UDP	DMVR	WP	OT
HGPP	8.2	4.8	5.9	18.2	40.1	6.1	16.7
EGPP	5.5	4.9	6.9	17.4	45.1	5.9	14.3

**Table 6:** Average processing times (in %) for each deblocking filter block profiled over the HGPP and EGPP.

	Conf.	CPLB	FD	LUF	CHF	OT
HGPP	AI	5.5	6.3	63.6	8.9	15.7
	RA	10.7	7.4	68.5	5.6	7.8
EGPP	AI	4.9	6.8	69.3	6.8	12.2
	RA	7.3	7.9	74.2	5.0	5.6

**Table 7:** Average processing times (in %) for the ALF block profiled over the HGPP and EGPP.

	Conf.	CRY	VBC	DeC	LCF	CHCF	CRCF	OT
HGPP	AI	8.3	1.4	17.5	37.7	10.7	22.9	1.5
	RA	12.6	1.4	21.6	43.2	8.0	11.6	1.6
EGPP	AI	0.8	0.8	10.7	66.4	16.6	3.9	0.8
	RA	1.1	0.8	11.8	72.1	11.5	1.9	0.8

**Table 8:** Comparison of the number of fps decoded by different VVC implementations.

Reference	Algorithm	Min–Max fps per Conf.		Hardware
		AI	RA	
Our HGPP	VTM8.0	0.6–2.3	1.2–4.1	Ryzen 3.4 GHz
Our EGPP	VTM8.0	0.3–0.8	0.4–1.2	ARM v8.2 2.2 GHz
[17]	VTM5.0	N/A	1.3–4.2	i9-9900X 3.5 GHz
[16]	VTM6.1	N/A	1.7–4.7	i7-8700K 4.7 GHz
[16] GPU	VTM6.1	N/A	2.1–8.4	RTX 2080Ti 1.6 GHz
[5]	VTM6.0	N/A	N/A	i7-4790k 4.0 GHz

**Table 9:** Comparison of the computational load distribution (in %) among functional blocks for different VVC decoder implementations.

	Reference	ED	TX	IP	EP	DBF	SAO	ALF	OT
AI	our HGPP	19	9	23	0	32	3	7	7
	our EGPP	18	8	22	0	24	1	19	8
	[5]	27	10	12	0	17	3	13	18
RA	our HGPP	9	5	6	33	26	2	11	8
	our EGPP	8	4	3	35	16	1	27	6
	[17]	3	2	12	41	19	3	19	-
	[16]	7	7	2	39	8	1	31	5
	[5]	9	3	2	33	17	3	13	10

**Table 10:** Optimistic (max) and pessimistic (min) estimation of the performance achieved in future optimized solutions, resulting speedup for those scenarios is also provided.

Platform	Scenario	Configuration	Average	Min	Max
HGPP	Un-optimized (fps)	AI	2.1	0.9	4.2
		RA	6.0	1.8	13.8
	Optimized (fps)	AI	8.4	4.0	16.3
		RA	33.6	10.9	66.5
	Speedup	AI	4.1	3.3	5.6
		RA	5.6	4.8	9.1
EGPP	Un-optimized (fps)	AI	0.9	0.4	1.8
		RA	1.8	0.6	3.9
	Optimized (fps)	AI	3.8	1.6	6.6
		RA	6.6	5.7	9.3
	Speedup	AI	3.8	3.2	4.9
		RA	6.6	5.7	9.0

## Data Availability

Contact with corresponding author.
